# Tolerogenic Programming Promotes Human Cytomegalovirus Permissiveness in Dendritic Cells

**DOI:** 10.1002/eji.70306

**Published:** 2026-10-28

**Authors:** Katharina Rahmel‐Stein, Jennifer Becker, Berenike Lange, Luise Krajewski, Britta Eiz‐Vesper, Ulrich Kalinke

**Affiliations:** ^1^ Institute for Experimental Infection Research, TWINCORE Centre for Experimental and Clinical Infection Research, a Joint Venture between the Helmholtz Centre for Infection Research and the Hannover Medical School Hannover Germany; ^2^ Institute for Transfusion Medicine and Transplant Engineering Hannover Medical School Hannover Germany; ^3^ Cluster of Excellence ‐ Resolving Infection Susceptibility (RESIST, EXC 2155) Hannover Medical School Hannover Germany

**Keywords:** antiviral immunity, HCMV infection, immune evasion, monocyte‐derived dendritic cells, tolerogenic dendritic cells, viral susceptibility

## Abstract

Dendritic cells (DCs) are important targets of human cytomegalovirus (HCMV), yet the factors determining their permissiveness to infection remain incompletely understood. We previously identified a CD1a^−^/CD86^−^ monocyte‐derived DC (moDC) subset with a tolerogenic transcriptional profile and increased susceptibility to HCMV. To test whether tolerogenic programming promotes DC permissiveness, we generated tolerogenic DCs (tolDCs) using vitamin D3 (VitD3), dexamethasone (Dexa), or their combination. TolDCs treated with Dexa alone or with VitD3+Dexa showed a maturation‐resistant phenotype, whereas VitD3‐treated cells retained partial responsiveness to maturation stimuli. Compared with conventional moDCs, tolDCs were more susceptible to HCMV infection. Functionally, HCMV‐exposed tolDCs induced markedly weaker CD4^+^ and CD8^+^ T‐cell proliferation, with Dexa‐treated cells showing the lowest stimulatory capacity. Together, these findings identify tolDCs as a permissive niche for HCMV and suggest that the virus may exploit regulatory DC states to evade immune control.

## Graphical abstract

Tolerogenic programming renders dendritic cells more permissive to HCMV infection and limits their capacity to stimulate T cells. These findings suggest that tolerogenic DCs serve as a permissive cellular niche that may facilitate HCMV immune evasion.

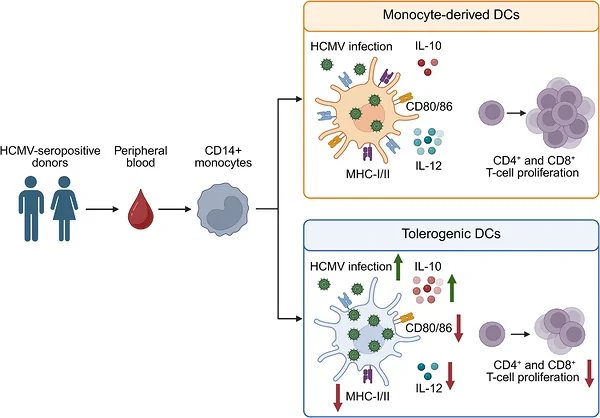

**Abbreviations**
DC: dendritic cell
GFP: green fluorescent protein
HCMV: human cytomegalovirus
hpi: hours postinfection
moDC: monocyte‐derived dendritic cell
SD: standard deviation
tolDC: tolerogenic dendritic cell

## 1 Introduction

Dendritic cells (DCs) are central regulators of antiviral immunity, linking innate sensing to priming and re‐stimulation of virus‐specific T cells [1, 2]. Accordingly, many viruses, including human cytomegalovirus (HCMV), manipulate DC functions to evade immune control and establish persistent infection [3, 4]. Myeloid cells, such as DCs, are key targets of CMV in vivo and contribute to viral dissemination and persistence [5, 6, 7].

In previous studies, we showed that although most monocyte‐derived DCs (moDCs) become infected after exposure to HCMV, only a minority of these cells initiate viral gene expression [8, 9]. Transcriptomic analysis further identified three distinct moDC subsets defined by differential expression of CD1a and CD86 [9], with CD1a^−^/CD86^−^ moDCs showing increased susceptibility to HCMV. Functional pathway analysis of selected DC‐related gene sets revealed that CD1a^−^/CD86^−^ moDCs expressed lower levels of genes involved in MHC‐I and MHC‐II antigen presentation, alongside high levels of CCL18, a chemokine linked with tolerogenic DC (tolDC) differentiation and regulatory T cell (Treg) induction [10]. Together, these findings suggest that increased viral permissiveness may be associated with a more tolerogenic DC phenotype.

TolDCs suppress pathogenic immune responses through inhibition of effector T‐cell activity, production of immunosuppressive cytokines, and induction of Tregs [11]. Although tolDCs have been studied mainly in the context of autoimmunity and transplantation, similar immunoregulatory DC states have been described also in infectious disease settings, where they may contribute to impaired antiviral immunity and viral persistence [12]. However, it remains unclear whether tolerogenic programming directly affects DC susceptibility to viral infection or whether viruses preferentially target such immunoregulatory cellular states to evade antiviral T‐cell responses.

Here we generated tolDCs using three different established in vitro differentiation protocols based on vitamin D3 (VitD3), dexamethasone (Dexa), or their combination (VitD3+Dexa) [13, 14, 15], and confirmed the tolerogenic phenotype of the respective tolDC subsets by spectral flow cytometry and multiplex cytokine analysis. We then assessed their susceptibility to HCMV infection relative to conventional moDCs and examined the functional consequences for T‐cell activation in co‐culture assays.

## 2 Results and Discussion

### 2.1 Vitamin D3‐ and Dexamethasone‐Treated Dendritic Cells Display a Maturation‐Resistant Tolerogenic Phenotype

Primary human monocytes were differentiated into donor‐matched conventional moDCs and by additional usage of vitamin D3 (VitD3), dexamethasone (Dexa), or their combination (VitD3+Dexa) into tolDCs (gating strategy in Figure S1B). The resulting populations were analyzed by spectral flow cytometry for the expression of surface markers associated with DC activation and maturation. Compared with moDC, tolDC cultures showed a modest reduction in cell recovery, while the overall viability was largely similar, with only a slight but statistically significant decrease observed in Dexa‐treated tolDCs (Figure S2A–C). Immature moDCs expressed low levels of CD80, CD83, and CD86, which were significantly upregulated upon maturation (Figure 1A–D), whereas CD40 and HLA‐DR remained largely unchanged (Figure S3C,D). Consistent with previous studies [16, 17], Dexa tolDCs exhibited a maturation‐resistant phenotype characterized by persistently low CD80, CD83, and CD86 expression (Figure 1A–E). In contrast, HLA‐DR expression was increased when compared with moDCs, but did not differ significantly between immature and mature Dexa tolDCs (Figure S3D). This divergent regulation of HLA‐DR and costimulatory molecules may reflect differential effects of Dexa treatment on antigen presentation and costimulatory pathways. VitD3+Dexa tolDCs showed a similarly stable phenotype, characterized by low CD83 expression and moderately elevated CD14 levels regardless of the maturation state (Figure 1C,E; Figure S3B), which was consistent with the reported retention of CD14 expression in VitD3‐treated tolDCs [18]. Unlike the other tolDC subsets, VitD3 tolDCs retained maturation responsiveness and upregulated CD83 and CD86 to levels comparable to those observed in conventional moDCs (Figure 1B–D; Figure S3A). Additional phenotypic profiling of DC‐ and tolDC‐associated surface markers revealed distinct expression patterns of CD1a, CCR7, ILT4, and PD‐L1 across the tolDC subsets tested, whereas CD11c expression was largely preserved (Figure S4A). Notably, the frequency of CD1a^+^ cells was reduced in the immature tolDC subsets. In contrast, maturation‐associated CCR7 upregulation was retained in VitD3 and VitD3+Dexa tolDCs but was largely absent in Dexa tolDCs. Cytokine analysis showed reduced IL‐12p70 secretion compared with moDCs, along with an increased IL‐10/IL‐12p70 ratio, particularly in Dexa and VitD3+Dexa tolDCs (Figure S4B), further supporting the less inflammatory and less activated phenotype of mature tolDCs. Together, these data confirm that different tolDC differentiation protocols generate phenotypically distinct DC populations, consistent with the previously described heterogeneity of tolDCs [13, 17]. However, canonical tolDC‐associated features, including increased ILT4 and PD‐L1 expression and resistance to maturation, were not observed across all conditions tested, highlighting protocol‐dependent differences and donor‐specific variation in tolerogenic programming.

**FIGURE 1:**
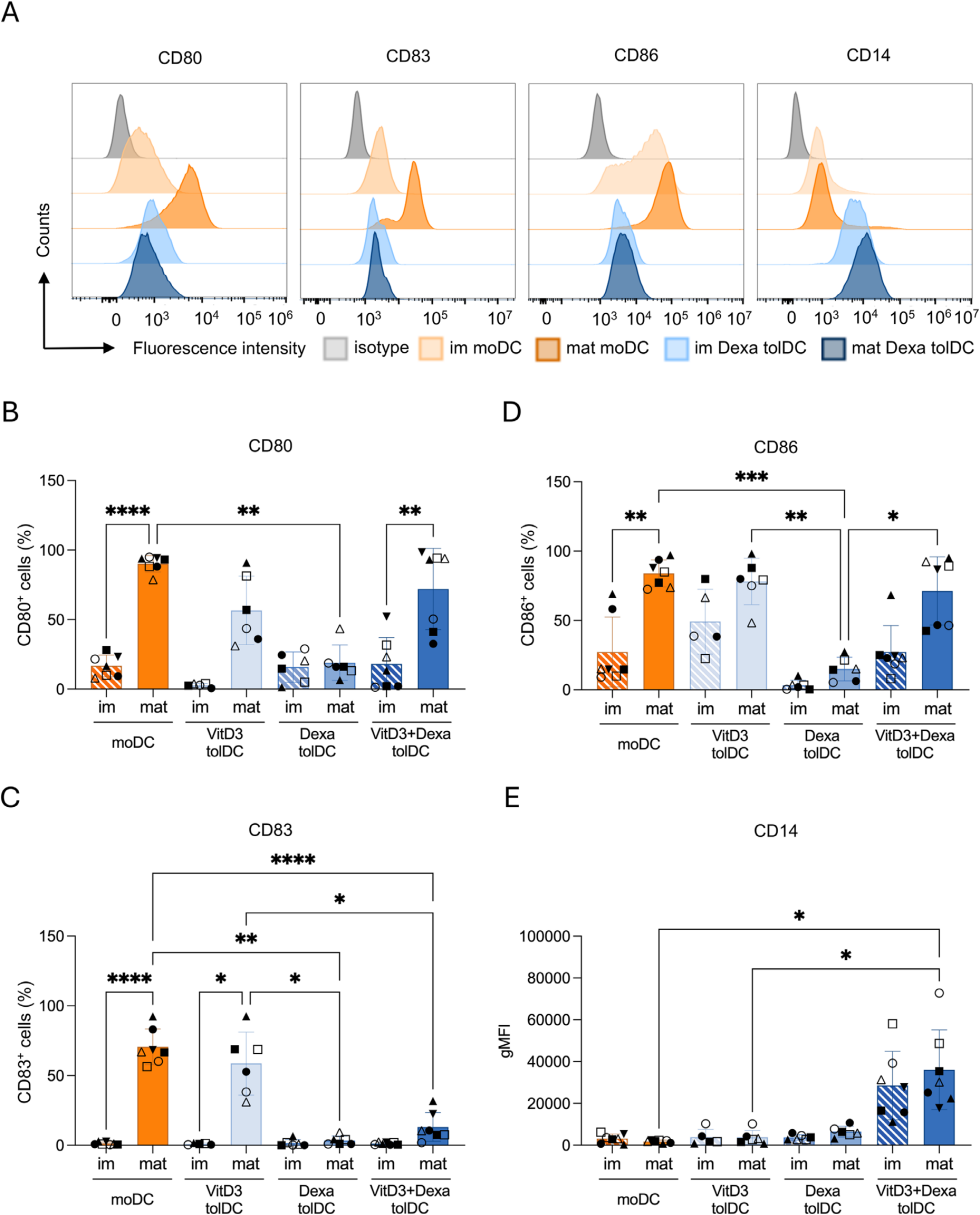
Vitamin D3‐ and dexamethasone‐treated DCs display a tolerogenic and maturation‐resistant phenotype. (A) Expression levels of CD80, CD83, CD86, and CD14 in immature moDCs (light orange), mature moDCs (orange), immature Dexa tolDCs (light blue), and mature Dexa tolDCs (dark blue) were analyzed by spectral flow cytometry. Individual histograms show the cell count and fluorescent intensity of indicated surface markers in moDC and tolDC populations after treatment with 1 µM dexamethasone for one representative donor. Isotype controls are depicted in grey. (B–D) Percentage of CD80‐, CD83‐, and CD86‐positive cells in immature versus mature moDC and tolDC populations. (E) The geometric mean fluorescent intensity (gMFI) of CD14 surface expression in moDCs and tolDCs was calculated for each sample, and nonspecific background fluorescence was corrected by subtracting the gMFI of the corresponding isotype control. Data are presented as mean ± SD of two independent experiments with a total of *n* = 6–7 donors. Each symbol represents one donor. Matched symbols within a figure indicate the same donor across experimental conditions. Symbol assignments are independent between figures and do not represent the same donor across different experiments. Statistical analysis was performed using a mixed‐effects model followed by Šidák's multiple comparisons test. All relevant comparisons were statistically assessed; comparisons without annotation did not reach statistical significance. *p < 0.05, **p < 0.01, ***p < 0.001, ****p < 0.0001, ns = not significant.

### 2.2 Tolerogenic Dendritic Cells Are More Susceptible to HCMV Infection Than Conventional Monocyte‐Derived Dendritic Cells

To assess the susceptibility of tolDCs to HCMV infection, immature moDCs and tolDCs were infected with green fluorescent protein (GFP)‐expressing HCMV at a multiplicity of infection (MOI) of 3 and analyzed by spectral flow cytometry 24 h postinfection (hpi) (gating strategy in Figure S1B). As reported earlier [9], conventional moDCs showed moderate susceptibility (17.2% GFP‐positive cells). In contrast, tolDCs exhibited markedly higher percentages of GFP‐positive cells, with VitD3+Dexa tolDCs being the most infected cell subset (62.3%), followed by VitD3 (38.9%) and Dexa tolDCs (34.8%) (Figure 2A). Pairwise donor analysis confirmed significantly higher permissiveness of Dexa and VitD3+Dexa tolDCs compared with moDCs (Figure 2B). Although overall GFP expression levels were similar to moDCs, HCMV‐exposed VitD3 tolDCs displayed moderately increased percentages of GFP‐positive cells in 7 of 8 donors (Figure 2B). Importantly, HCMV infection did not negatively correlate with cell viability (Figure S2D), indicating that reduced viability was not a confounding factor. Consistent with the increased HCMV‐GFP reporter expression at 24 hpi, culture supernatants from VitD3 and VitD3+Dexa tolDCs collected at 6 and 8 days postinfection contained higher levels of infectious viral progeny than those from donor‐matched conventional moDCs (Figure 2C). No such increase was observed in Dexa tolDCs, potentially due to their reduced viability. Together, these data show that the enhanced permissiveness of VitD3‐treated tolDCs is accompanied by increased production of infectious progeny, thus confirming productive HCMV infection. Despite their enhanced HCMV permissiveness, HCMV‐exposed tolDCs retained a less activated phenotype than conventional moDCs. Expression of CD40, CD80, CD86, HLA‐ABC, and HLA‐DR was generally lower, with the extent of downregulation varying across markers and tolDC subsets (Figure 2D). Notably, CD86 was significantly reduced across all three tolDC populations, and HLA‐ABC expression was significantly lower in VitD3 and VitD3+Dexa tolDCs. This pattern is consistent with our previous finding that the highly HCMV‐permissive CD1a^−^/CD86^−^ moDC subset exhibited reduced expression of genes involved in DC activation and antigen presentation, together with high expression of the tolerogenic chemokine CCL18 [9]. In summary, these observations suggest that tolDCs, particularly those generated with VitD3+Dexa, are substantially more permissive to HCMV infection than conventional moDCs. Accordingly, enhanced HCMV susceptibility appears to be linked with tolerogenic programming rather than being restricted to a single DC subset. Analysis of HCMV entry‐associated surface molecules revealed increased CD141 in VitD3 and VitD3+Dexa tolDCs and increased HLA‐ABC in Dexa tolDCs, whereas CD209 was reduced in Dexa and VitD3+Dexa tolDCs, and CD61 remained comparable to moDCs (Figure S5). Although no common expression pattern was observed across tolDC subsets, distinct entry factors, together with differences in intracellular signaling pathways or antiviral responses, may contribute to their increased HCMV permissiveness [13, 19].

**FIGURE 2:**
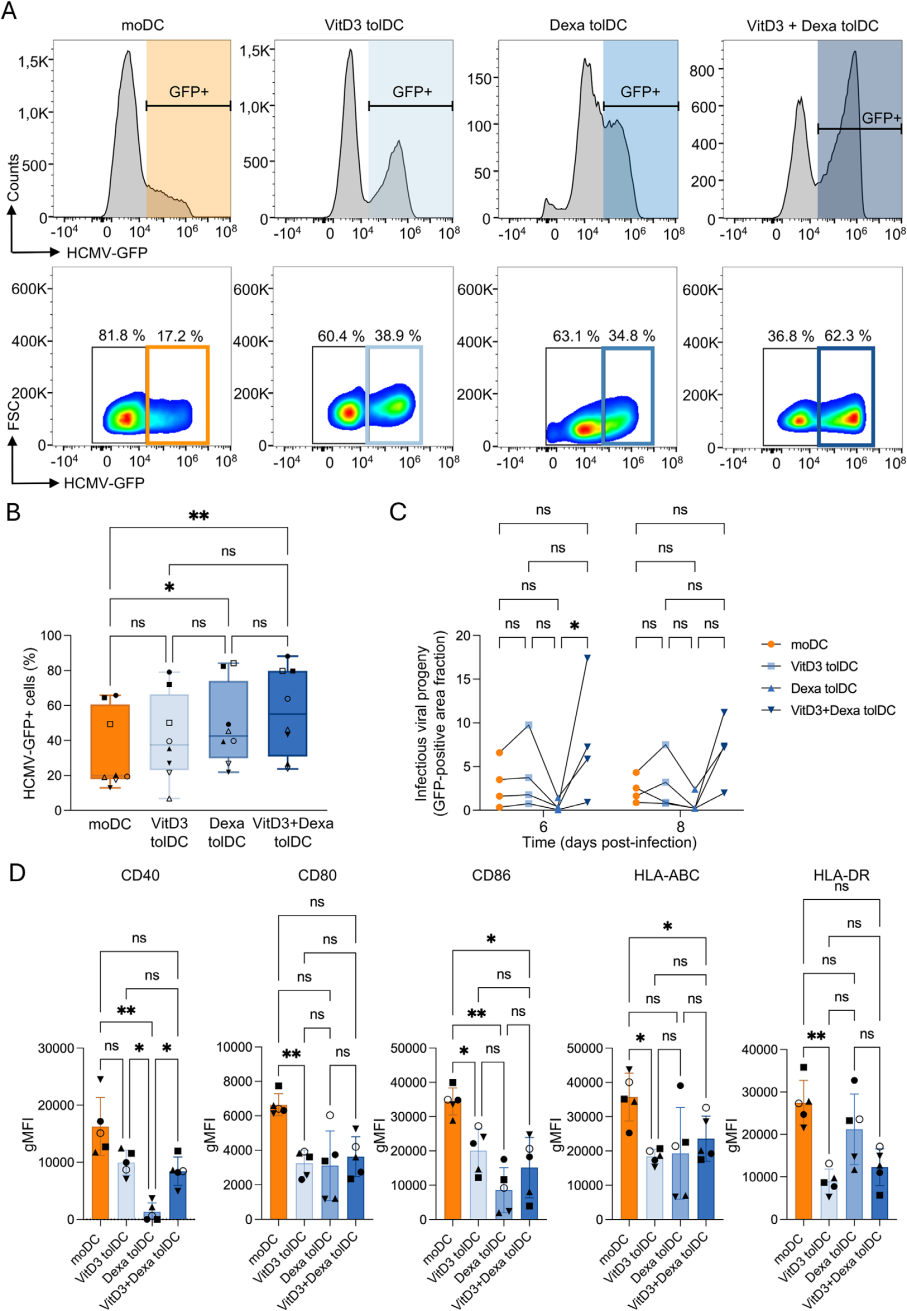
tolDCs display higher infection susceptibility but reduced activation compared with moDCs at 24 hpi. moDCs and tolDCs were infected with HCMV‐GFP at MOI 3 for 24 h. Subsequently, cells were harvested and analyzed for GFP expression by flow cytometry. (A) Representative histograms (top panel) and corresponding dot plots (bottom panel) showing GFP expression in moDCs, VitD3 tolDCs, Dexa tolDCs, and VitD3+Dexa tolDCs. Percentages of GFP‐positive and GFP‐negative cells are indicated for one representative donor. (B) Statistical analysis of the change in percentages of GFP‐positive moDCs compared with tolDCs. (C) Measurement of infectious viral progeny in supernatants of HCMV‐infected moDCs and tolDCs at 6 and 8 days postinfection. (D) Surface expression levels of CD40, CD80, CD86, HLA‐ABC and HLA‐DR on HCMV‐infected moDCs and tolDCs were determined by flow cytometry and are presented as geometric mean fluorescence intensity (gMFI). Data are shown as mean ± SD of *n* = 7–8 (B), *n* = 4 (C), and *n* = 5 (D) independent donors. Each donor is represented by a different symbol. Statistical analyses were performed using a Friedman test followed by Dunn's multiple comparisons test in (B) and a repeated‐measures (RM) one‐way analysis of variance (ANOVA) followed by Tukey's multiple comparisons test in (C, D). *p < 0.05, **p < 0.01, ns = not significant.

### 2.3 HCMV‐Exposed Tolerogenic Dendritic Cells Show Reduced T‐Cell Stimulatory Capacity, and May Contribute to Immune Evasion

To determine whether increased viral susceptibility affects the immunogenicity of tolDCs, HCMV‐exposed moDCs and tolDCs were co‐cultured with autologous CD4^+^ and CD8^+^ T cells from HCMV‐positive donors, and T‐cell proliferation was quantified by CellTrace Yellow dilution (Figure 3A; Figure S1C). Unstimulated DCs did not induce T‐cell proliferation, whereas HCMV‐exposed moDCs elicited robust CD4^+^ and CD8^+^ T‐cell responses (up to 60% and 83% proliferation, respectively) (Figure 3B,C). In contrast, HCMV‐exposed tolDCs induced significantly weaker proliferation (<45% in CD4^+^ T cells and <55% in CD8^+^ T cells). Among tolDC subsets, Dexa‐treated cells showed the lowest stimulatory capacity, followed by VitD3+Dexa tolDCs, while T‐cell responses in VitD3 tolDC co‐cultures were significantly reduced for CD4^+^ but not CD8^+^ T cells, when compared with moDCs (Figure 3B,C). This reduced T‐cell stimulation capacity is consistent with the maturation‐resistant phenotype of tolDCs and the reduced expression of costimulatory and antigen‐presentation molecules in HCMV‐infected tolDCs compared with infected moDCs. Notably, despite higher frequencies of GFP‐positive cells, HCMV‐exposed tolDCs induced less T‐cell proliferation than donor‐matched moDCs, indicating that increased infection did not translate into enhanced T‐cell stimulation. Similar effects have been reported in studies investigating tolDC‐based cellular therapies, where IL‐10‐transduced or VitD3‐conditioned DCs showed reduced capacity to induce antigen‐specific effector T‐cell responses [20, 21]. Collectively, these findings suggest that tolerogenic programming, together with the phenotypic changes induced by HCMV, contributes to the reduced T‐cell stimulatory capacity of tolDCs, as reflected by diminished T‐cell proliferation. The relative contribution of altered maturation, cytokine production, and antigen presentation, however, remains to be determined.

**FIGURE 3:**
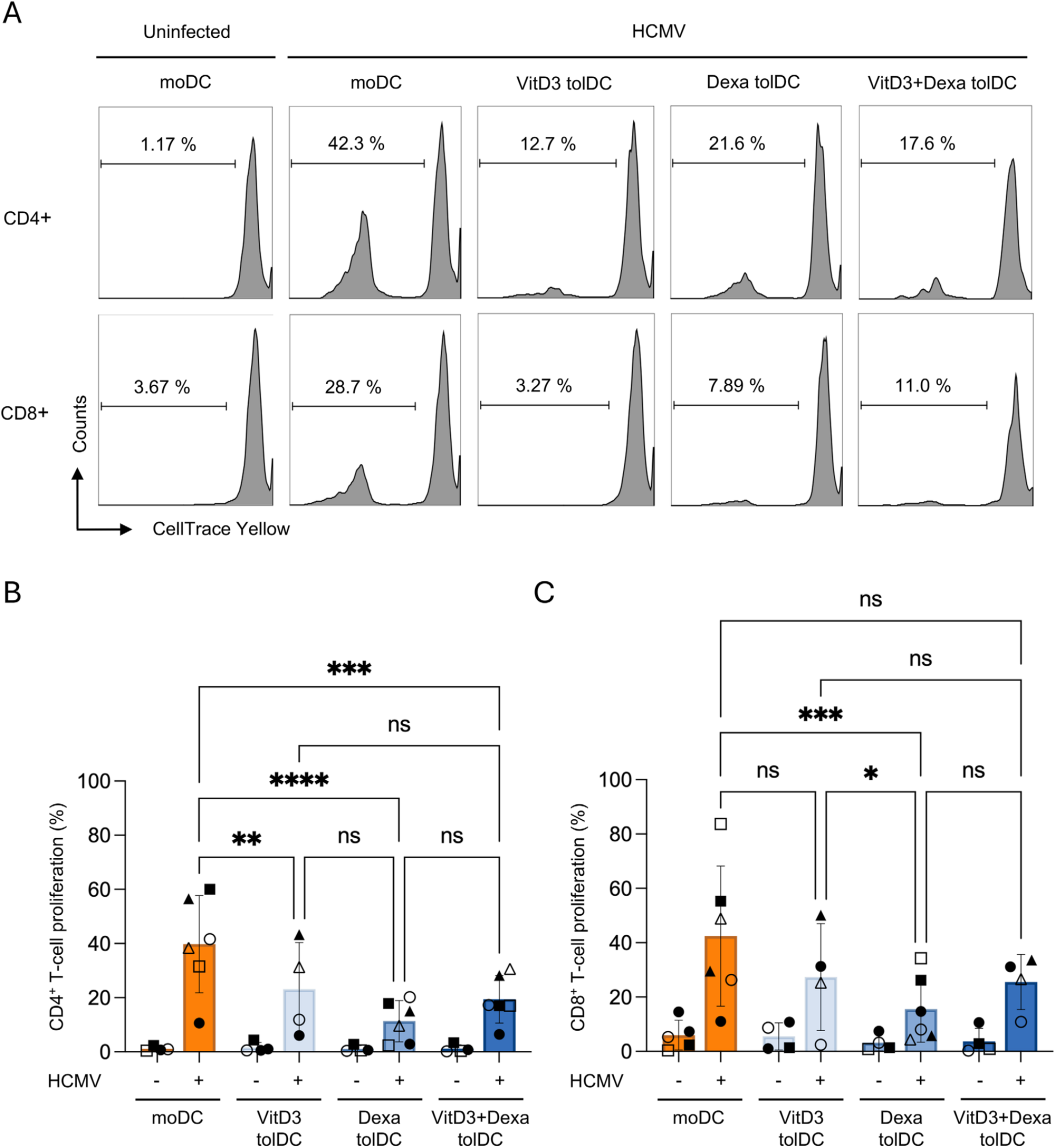
After HCMV exposure, tolDCs induce less CD4^+^ and CD8^+^ T‐cell proliferation than moDCs. moDCs, VitD3 tolDCs, Dexa tolDCs, and VitD3+Dexa tolDCs were exposed to HCMV‐GFP at MOI 3 for 24 h. Then, uninfected or HCMV‐infected DCs were co‐cultured with autologous CD3^+^ T cells at an effector‐to‐target ratio of 10:1 (T cell:DC). After 7 days, co‐cultures were harvested, immune‐labeled with antibodies against CD3, CD4, and CD8, and analyzed by flow cytometry. (A) T‐cell proliferation was assessed using CellTrace Yellow staining. Histograms show the percentage of proliferating CD4^+^ (top panel) and CD8^+^ (bottom panel) T cells for one representative donor. (B) CD4^+^ and (C) CD8^+^ T‐cell proliferation frequencies following co‐culture with uninfected (−) or HCMV‐infected (+) moDCs and tolDCs. Data were pooled from two independent experiments. Bar diagrams show the mean ± SD for *n* = 4–6 independent donors. Each donor is represented by a different symbol. Identical symbols across experimental conditions represent the same donor. Donors are identical to those shown in Figure 2, with consistent symbol assignments in both figures. Statistical analysis was performed using a two‐factor mixed‐effects model (REML) followed by Tukey‘s multiple comparisons test for pairwise comparisons between DC conditions. *p < 0.05, **p < 0.01, ***p < 0.001, ****p < 0.0001, ns = not significant.

Several persistent pathogens, including HIV‐1 [22], exploit immature or tolerogenic DCs to weaken antiviral immunity. Similarly, HCMV encodes immunomodulatory factors that disrupt DC function, such as the viral IL‐10 homolog cmvIL‐10 [23] and soluble CD83 [24], both of which impair DC‐mediated T‐cell activation. Preferential infection of tolDCs may therefore represent an immune evasion mechanism that subverts DC‐driven immunity by selectively targeting DC populations with intrinsically lower immunogenicity.

Given the role of tolDCs in promoting immune tolerance, including through the induction of Tregs [11], HCMV infection of these cells may further skew immune responses toward regulation rather than protective immunity. Consistent with this concept, studies with murine cytomegalovirus have shown that Tregs can suppress antiviral CD8+ T‐cell responses, resulting in increased viral loads during acute infection and the establishment of latent persistence in the spleen [25, 26]. However, whether HCMV‐infected tolDCs similarly drive the expansion of Tregs remains to be determined. A better understanding of how HCMV exploits immunoregulatory cell states may eventually enable the development of strategies to selectively target highly permissive DC subsets and limit viral persistence without broadly compromising immune tolerance.

## 3 Material and Methods

### 3.1 Primary Cell Isolation

Blood samples of healthy platelet donors were obtained from the Institute of Transfusion Medicine and Transplant Engineering (Hannover Medical School, Germany) following written informed consent (ethical approval no. 8315_BO_K_2019). Peripheral blood mononuclear cells (PBMCs) were isolated using density gradient centrifugation.

### 3.2 In Vitro Differentiation of Monocyte‐Derived and Tolerogenic Dendritic Cells

CD14^+^ monocytes were purified by magnetic separation (Miltenyi Biotec) and differentiated into immature moDCs in CellGenix GMP DC medium supplemented with 1000 U/mL IL‐4 and 1000 U/mL GM‐CSF (Miltenyi Biotec) at 37°C for 5 days. DC maturation was induced on day 5 using 10 ng/mL TNF‐α, 10 ng/mL IL‐1β, 1000 U/mL IL‐6, and 1 µg/mL PGE2 for 48 h (Figure S1A). TolDCs were obtained by treating monocyte cultures either with 1 nM 1α,25‐dihydroxyvitamin D3 (Sigma‐Aldrich) or 1 µM dexamethasone (Sigma‐Aldrich) on day 0 and 4, or with a combination of 100 nM 1α,25‐dihydroxyvitamin D3 and 10 nM dexamethasone on day 5 as previously described [13, 14, 15]. TolDCs were cultured and matured under the same conditions as moDCs.

### 3.3 HCMV Infection

Recombinant HCMV expressing GFP (HCMV strain RV‐TB40‐BAC_KL7_‐SE‐EGFP UL40rep‐delUS2‐6+US11 [27], referred to as HCMV‐GFP) was propagated in MRC‐5 lung fibroblasts (ATCC CCL‐171) and purified by density gradient centrifugation. Infectious virus yield was determined on MRC‐5 cells as previously described [28]. MoDCs or tolDCs were infected with HCMV‐GFP on day 6 (MOI 3). To enhance the efficiency of infection, cells were centrifuged for 30 min at 300×*g*. HCMV‐exposed moDCs and tolDCs were analyzed at 24 hpi by quantifying the percentage of cells expressing the virally encoded GFP by flow cytometry, or they were used for T‐cell co‐culture assays.

To assess infectious virus release, supernatants from HCMV‐infected moDC and tolDC cultures were collected at 6 and 8 days postinfection, centrifuged to remove cell debris, and transferred to MRC‐5 fibroblasts. After 2 days, infection of fibroblasts was quantified by determining the GFP‐positive area fraction using Fiji (ImageJ).

### 3.4 Isolation of Pan T Cells, Proliferation Staining, and Co‐Culture Assays

Autologous CD3^+^ T cells were isolated from frozen PBMCs of HCMV‐positive donors using the Pan T cell Isolation Kit (Miltenyi Biotec), labeled with CellTrace Yellow (Invitrogen), and plated in 96‐well round‐bottom plates at a density of 2 × 10^5^ cells/well. After 24 h, rested CD3^+^ T cells were cultured with uninfected or HCMV‐exposed moDCs or tolDCs at an effector‐to‐target ratio (T cell:DC) of 10:1. After 7 days, CD4^+^ and CD8^+^ T‐cell proliferation was analyzed by tracking the dilution of the fluorescent proliferation dye via flow cytometry.

### 3.5 Flow Cytometry Analysis

Cells were stained with Zombie NIR fixable live/dead stain (BioLegend) in cold PBS to identify dead cells. Fc receptors were blocked with human polyglobin and cells were immunolabeled with fluorochrome‐conjugated monoclonal antibodies against human CD1a, CD14, CD61 (all BD Biosciences), CD11c, CD40, CD80, CD83, CD86, CD141, CD209, CCR7, HLA‐ABC, HLA‐DR, ILT4, and PD‐L1 (all BioLegend) or against CD3, CD4, and CD8 (all BioLegend). Surface labeling was performed for 20 min at 4°C. Then, cells were washed with labeling buffer and fixed with 1% paraformaldehyde. Data were acquired on the ID7000 Spectral Cell Analyzer (SONY Biotechnology) and analyzed using FlowJo (version 10.10.0).

### 3.6 Multiplex Cytokine Assay

IL‐10 and IL‐12p70 concentrations were quantified in culture supernatants collected on day 7 using the LEGENDplex Human Inflammation Panel 1 (13‐plex) (BioLegend) according to the manufacturer's instructions. Samples were acquired as described above and analyzed with the LEGENDplex software (version 2026‐07‐01; BioLegend).

### 3.7 Statistical Analysis

Statistical analyses were performed in GraphPad Prism version 10.4.1 (GraphPad Software). Differences between groups were generally assessed using repeated‐measures one‐way analysis of variance (ANOVA) for complete paired datasets and mixed‐effects analysis (REML) for datasets with missing values. For the comparison of GFP‐positive cell frequencies between moDCs and tolDCs, a Friedman test was used due to the nonnormal distribution of the data. Correlation analyses were performed using simple linear regression with 95% confidence intervals. Statistical tests used for each analysis are indicated in the corresponding figure legends. A *p*‐value of < 0.05 was considered statistically significant.

## Author Contributions

Conceptualization: K.R.S., J.B., U.K. Formal analysis: K.R.S., J.B. Funding acquisition: J.B., U.K. Investigation: K.R.S., B.L. Methodology: K.R.S., J.B., L.K. Resources: L.K., B.E.V. Visualization: K.R.S., J.B. Writing – original draft: K.R.S., U.K. Writing – review and editing: all authors.

## Conflicts of Interest

The authors declare no conflicts of interest.

## Ethics Statement

The study was approved by the Ethics Committee of Hannover Medical School (approval no. 8315_BO_K_2019) prior to its initiation. Written informed consent was obtained from all donors before blood sample collection.

## Supporting information




**Supporting File**: eji70306‐sup‐0001‐Figures.pdf.

## Data Availability

The data that support the findings of this study are available in the article and its supporting information files. Additional raw data are available from the corresponding author upon reasonable request.
