# Supplementary material for: Tolerogenic Programming Promotes Human Cytomegalovirus Permissiveness in Dendritic Cells
Source: Eur J Immunol. 2026 Oct 28;56(10):e70306. doi: 10.1002/eji.70306 (PMC13714553; doi:10.1002/eji.70306)
Supplement: Supplementary file 1 [file EJI-56-e70306-s001.pdf]

SUPPORTING MATERIAL

Tolerogenic programming promotes human cytomegalovirus permissiveness in dendritic cells

Katharina Rahmel-Stein, Jennifer Becker, Berenike Lange, Luise Krajewski, Britta Eiz-Vesper, Ulrich Kalinke

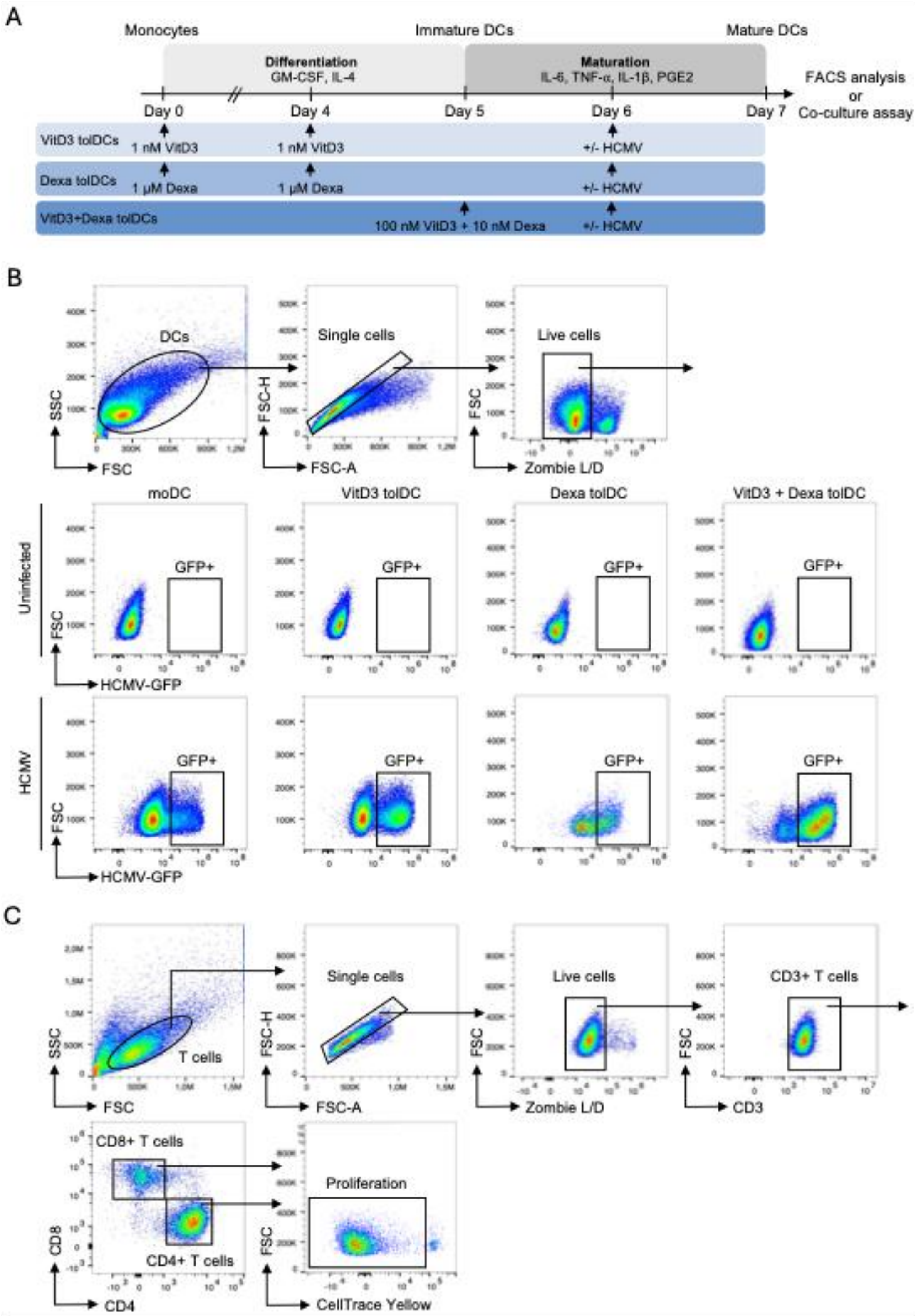

Supplementary Figure 1: Experimental workflow and flow cytometry gating strategies for DC and T-cell analyses. (**A**) Schematic representation of the *in vitro* generation, maturation and HCMV infection of moDCs and tolDCs. $CD14^+$ monocytes were isolated from PBMCs of healthy donors and differentiated with GM-CSF and IL-4 for 5 days. TolDCs were generated by additional treatment with vitamin D3 (VitD3), dexamethasone (Dexa) or their combination (VitD3+Dexa) as indicated. On day 5, immature DCs were either left untreated or stimulated with a maturation cocktail containing IL-6, TNF-α, IL-1β and PGE2 for 48 h. For infection experiments, DCs were treated with HCMV-GFP at MOI 3 on day 6. After 24 h, cells were harvested and analyzed by flow cytometry or they were co-cultured with autologous T cells. (**B**) Representative gating strategy for the analysis of moDCs and tolDCs.

Following identification of the DC population, single and live cells were selected. GFP^+ populations were identified based on GFP expression, with corresponding uninfected controls in the middle panel and HCMV-exposed samples in the lower panel. (**C**) Representative gating strategy for T-cell proliferation analysis following co-culture with moDCs or tolDCs. Single, live $CD3^+$ T cells were identified and separated into $CD4^+$ and $CD8^+$ T-cell populations. Proliferating cells were identified based on CellTrace Yellow dilution.

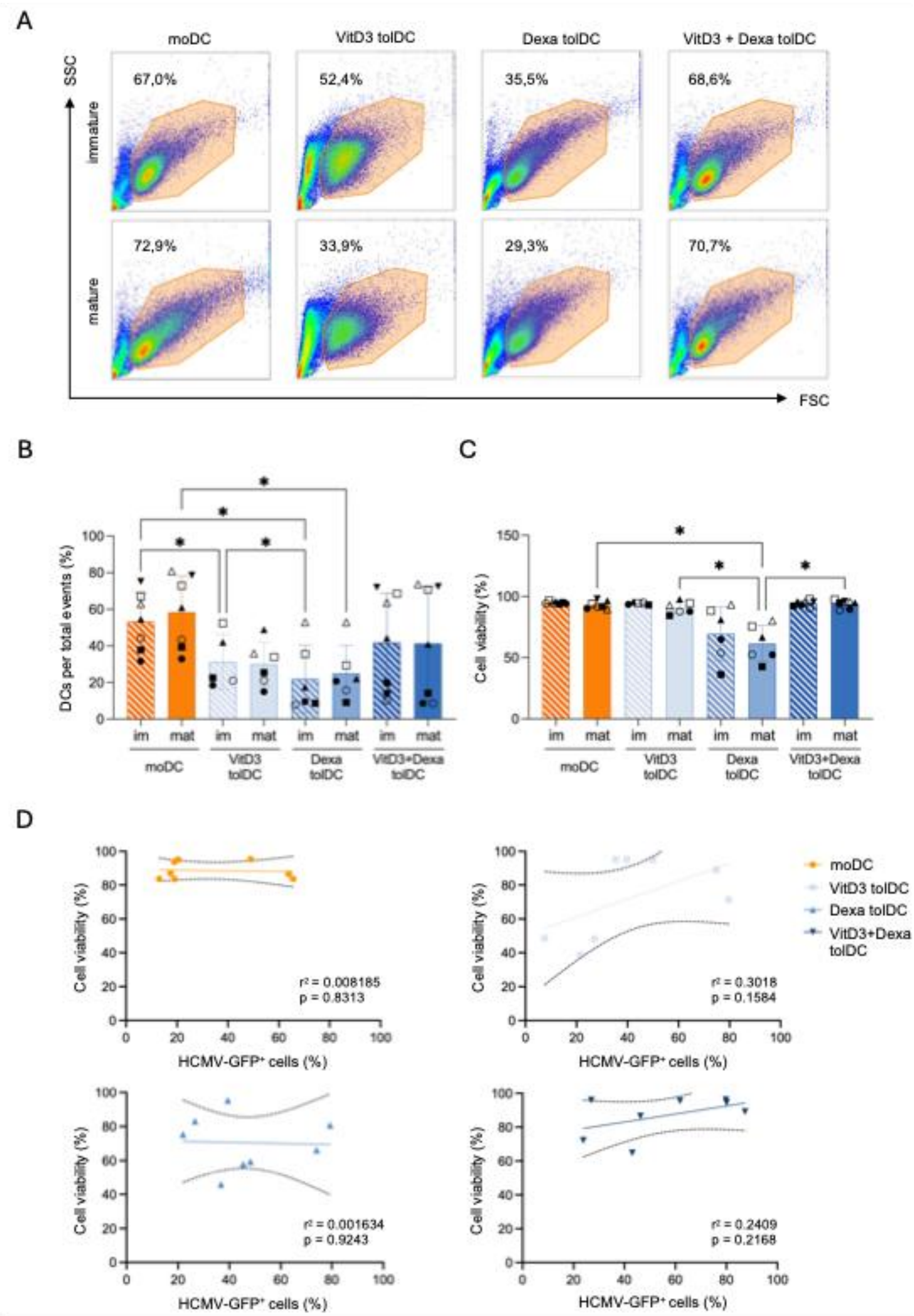

Supplementary Figure 2: Viability is comparable between moDCs and VitD3 or VitD3+Dexa tolDCs in both immature and mature states and does not correlate with the frequency of GFP-positive cells. (**A**) Representative forward and side scatter plots showing the gating strategy used to identify immature and mature moDCs and tolDCs. Data from one representative donor are shown. Percentages indicate the frequency of gated populations for each treatment condition. (**B**) Frequency of moDCs and tolDCs as percentage of total events. (**C**) Cell viability was assessed as the percentage of live cells using Zombie NIR live/dead staining. Data are presented as mean ± SD of two independent experiments with a total of n = 6-7 donors. Each donor is represented by a different symbol. Matched symbols within a figure indicate the same donor across experimental conditions.

Statistical differences were analyzed using a mixed-effects model followed by Šidák's multiple comparisons test. All relevant comparisons were statistically assessed; comparisons without annotation did not reach statistical significance. *$p < 0.05$. (**D**) Correlation analysis between cell viability (x-axis) and the percentage of HCMV-positive cells (y-axis) by simple linear regression with 95% confidence intervals. Coefficients of determination (r^2) and p-values are indicated. Treatment groups are distinguished by color and symbol.

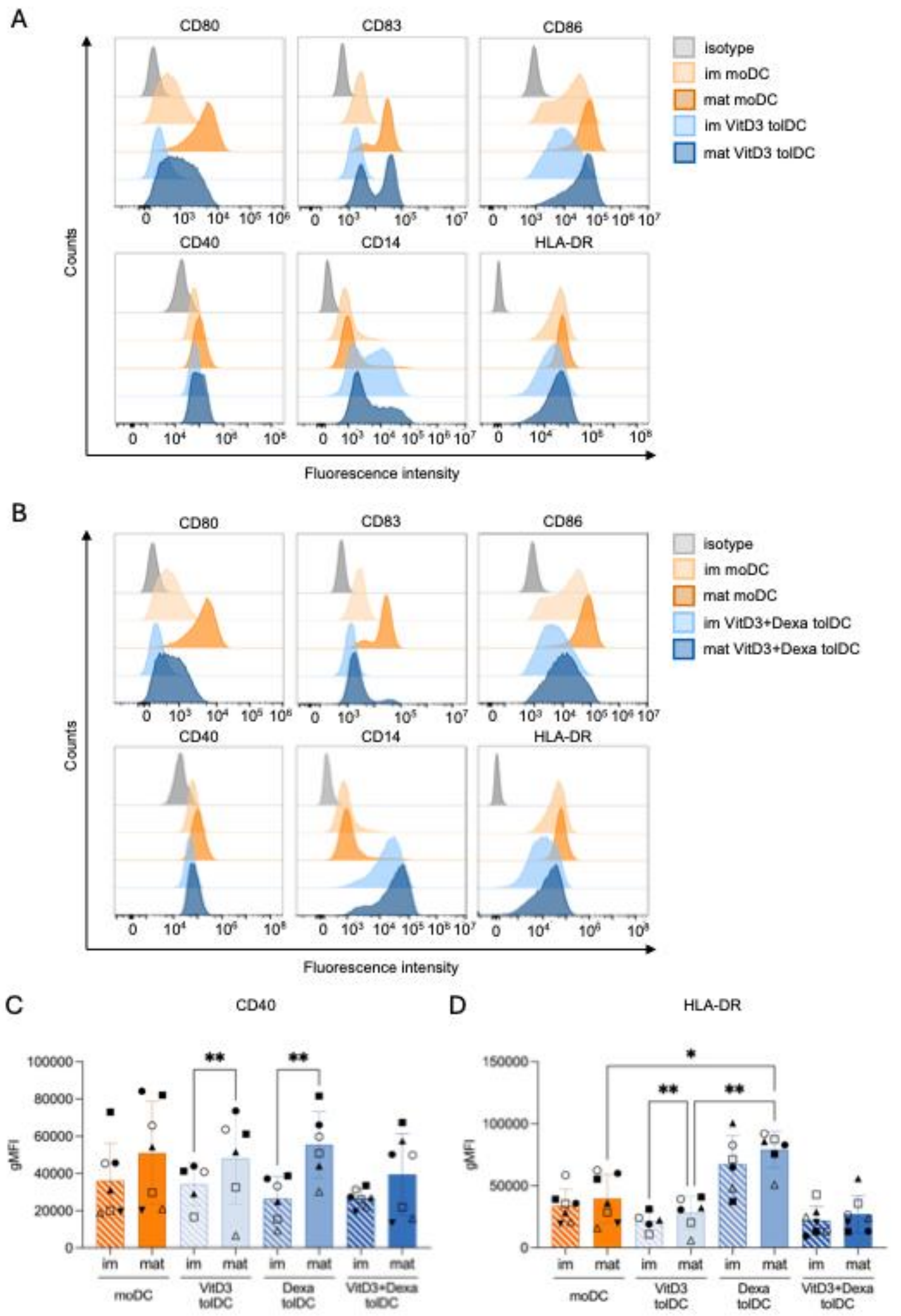

Supplementary Figure 3: TolDCs generated in the presence of dexamethasone express reduced levels of CD83 and CD86. Flow cytometric analysis of CD80, CD83, CD86, CD40, CD14 and HLA-DR on moDCs and tolDCs treated with vitamin D3 (**A**) or vitamin D3 and dexamethasone (**B**). Histograms show data of one representative donor. Isotype controls are depicted in grey. Statistical analysis of the geometric mean fluorescence intensity (gMFI) of CD40 (**C**) and HLA-DR (**D**) surface expression. Data are presented as mean ± SD of two independent experiments with a total of n = 6-7 donors. Each symbol represents one donor. Matched symbols within a figure indicate the same donor across experimental conditions Statistical analysis was performed using a mixed-effects model followed by Šidák‘s multiple comparisons test. All relevant comparisons were statistically assessed; comparisons without annotation did not reach statistical significance. *p < 0.05, **p< 0.01.

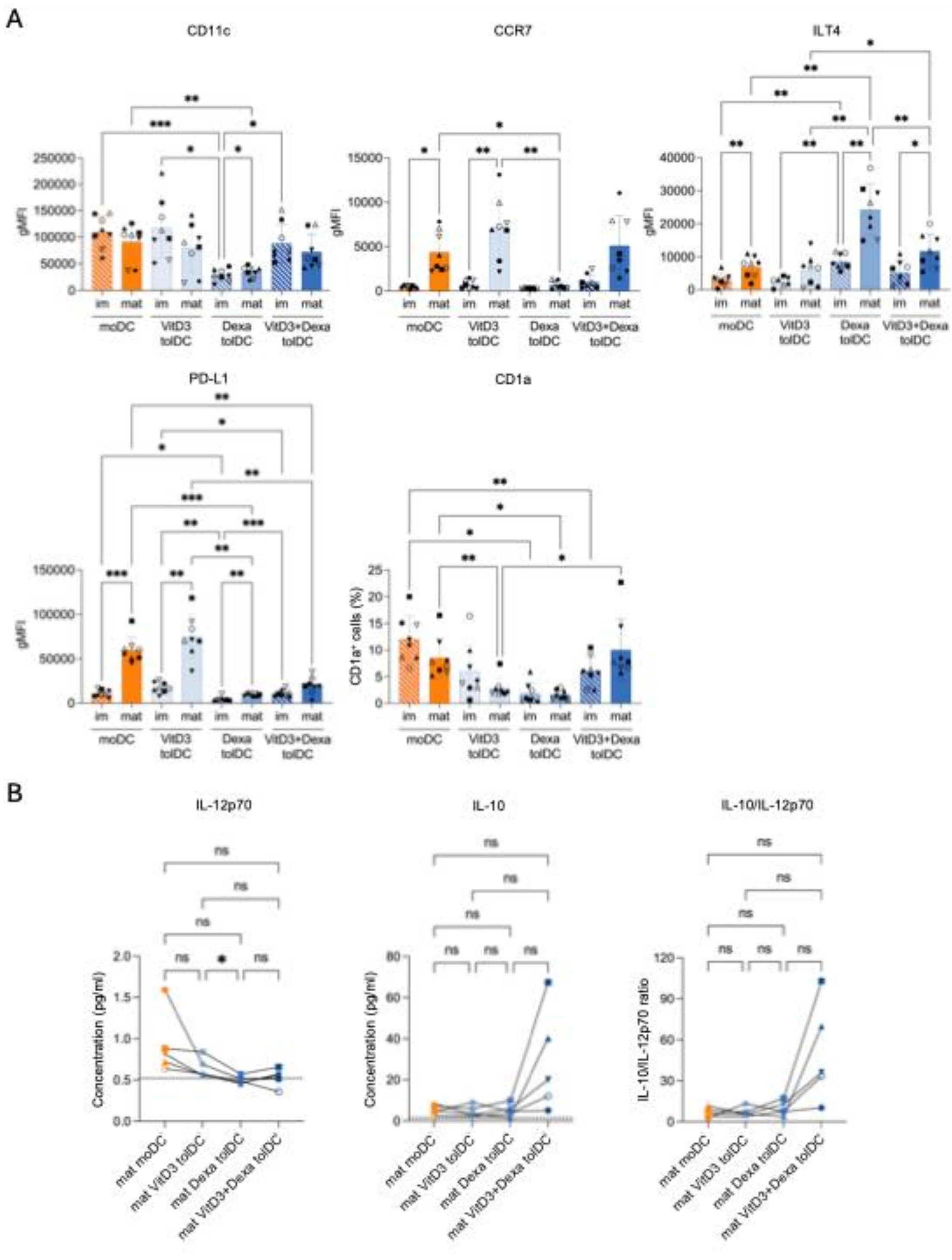

Supplementary Figure 4: Tolerogenic programming alters the phenotype and cytokine profile of moDCs. Immature and mature moDCs and tolDCs generated using vitamin D3 (VitD3), dexamethasone (Dexa) or their combination (VitD3+Dexa) were analyzed for CD11c, CCR7, ILT4, PD-L1 and CD1a expression by flow cytometry. (**A**) Data are shown as geometric mean fluorescence intensity (gMFI) for CD11c, CCR7, ILT4, and PD-L1 and as the percentage of CD1a⁺ cells. Individual donors are represented by distinct symbols, with matching symbols indicating the same donor across conditions. Data are presented as mean ± SD of n = 8 donors from two independent experiments. (**B**) IL-12p70 and IL-10 concentrations (pg/ml) in culture supernatants of mature moDCs and tolDCs were quantified using a LEGENDplex bead-based immunoassay and the IL10/IL12p70 ratio was calculated. Individual donors (n = 5) are represented by distinct symbols and connected by lines across conditions. Statistical differences were analyzed by repeated-measures one-way analysis of variance (ANOVA) followed by Šidák's multiple comparisons test (**A**) or Tukey's multiple comparisons test (**B**). *p < 0.05, **p< 0.01, ***p< 0.001, ns = not significant.

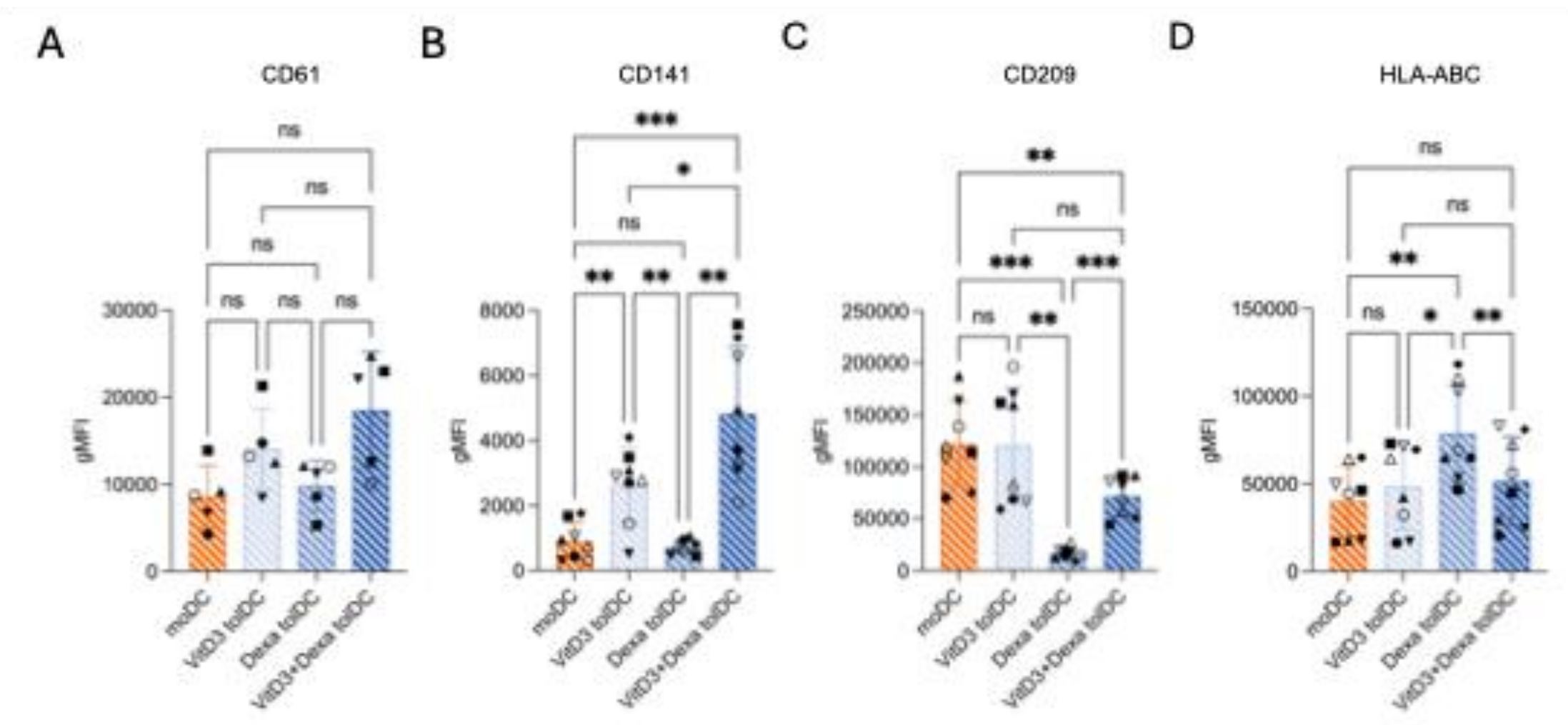

Supplementary Figure 5: Increased HCMV permissiveness of tolDCs does not correlate with a general upregulation of viral entry-associated molecules. Surface expression of HCMV entry-associated molecules was assessed in immature moDCs and tolDCs generated using vitamin D3 (VitD3), dexamethasone (Dexa) or their combination (VitD3+Dexa) by flow cytometry. Expression of (**A**) CD61 (n=5), (**B**) CD141, (**C**) CD209, and (**D**) HLA-ABC (n=8) is shown as geometric mean fluorescence intensity (gMFI). Individual donors are represented by distinct symbols, with matching symbols indicating the same donor across conditions. Data were obtained from two independent experiments and are presented as mean ± SD. Statistical differences were analyzed by repeated-measures one-way analysis of variance (ANOVA) followed by Tukey's multiple comparisons test. *p < 0.05, **p< 0.01, ***p< 0.001, ns = not significant.
